# New Insights into the Taxonomy of Bacteria in the Genomic Era and a Case Study with Rhizobia

**DOI:** 10.1155/2022/4623713

**Published:** 2022-05-21

**Authors:** Luisa Caroline Ferraz Helene, Milena Serenato Klepa, Mariangela Hungria

**Affiliations:** ^1^Embrapa Soja, CP 4006, 86085-981 Londrina, PR, Brazil; ^2^Conselho Nacional de Desenvolvimento Científico e Tecnológico, SHIS QI 1 Conjunto B, Blocos A, B, C e D, Lago Sul, 71605-001 Brasília, DF, Brazil; ^3^Department of Microbiology, Universidade Estadual de Londrina, CP 10011, 86057-970 Londrina, PR, Brazil; ^4^Coordenação de Aperfeiçoamento de Pessoal de Nível Superior, SBN, Quadra 2, Bloco L, Lote 06, Edifício Capes, 70040-020 Brasília, DF, Brazil

## Abstract

Since early studies, the history of prokaryotes taxonomy has dealt with many changes driven by the development of new and more robust technologies. As a result, the number of new taxa descriptions is exponentially increasing, while an increasing number of others has been subject of reclassification, demanding from the taxonomists more effort to maintain an organized hierarchical system. However, expectations are that the taxonomy of prokaryotes will acquire a more stable status with the genomic era. Other analyses may continue to be necessary to determine microbial features, but the use of genomic data might be sufficient to provide reliable taxa delineation, helping taxonomy to reach the goal of correct classification and identification. Here we describe the evolution of prokaryotes' taxonomy until the genomic era, emphasizing bacteria and taking as an example the history of rhizobia taxonomy. This example was chosen because of the importance of the symbiotic nitrogen fixation of legumes with rhizobia to the nitrogen input to both natural ecosystems and agricultural crops. This case study reports the technological advances and the methodologies used to classify and identify bacterial species and indicates the actual rules required for an accurate description of new taxa.

## 1. Introduction

The taxonomy terminology has been broadly discussed. Some researchers consider the taxonomy as systematics, while others define taxonomy as the classification of organisms and part of the systematics, which would have a broader scope, including studies with evolutionary and phylogenetic components [1]. Taxonomy is the science responsible for the orderly arrangement of living organisms respecting a hierarchical system that can presume the evolutionary relationships; it also offers relevant information about the origin of life and how it evolved on Earth [2, 3]. The objective of general taxonomy is to establish a classification system based on genealogical relationships, aiming to reach a natural system that mirrors the “order in nature” [4–6].

Prokaryotes include living organisms belonging to both domains, Archaea and Bacteria, known as archaebacteria or archaea and eubacteria, respectively. Those microorganisms do not have a distinct nucleus or other organelles due to the lack of internal membranes, main characteristics distinguishing them from the eukaryotes. The prokaryotic taxonomy is traditionally split into three correlated areas: classification, nomenclature, and identification [7, 8]. The orderly arrangement of organisms into taxonomic ranks designates the classification. The nomenclature objective is to name the organisms following the International Code of Nomenclature of Prokaryotes (ICNP) rules. The identification involves allocating the strains into the described taxonomic groups [7, 9, 10]. However, if the taxonomy of a strain remains undefined, the characterization and phylogenetic analysis should be carefully carried out to describe and name the new taxon.

The basic unit of taxonomy is the species. Bergey's Manual of Systematic Bacteriology defines a bacterial species as a group of strains with certain distinctive features that generally resemble each other in essential features of an organization [11]. Estimates are of about 1012 bacterial species on Earth [12]. In November 2021, there were about 17,845 valid species names (without synonyms) [13]. Considering this number, we can conclude that bacterial diversity is not sufficiently explored and that most might still be composed of still uncultivable species.

Besides arranging the organisms, the taxonomic tools are used to study microbial diversity and establish phylogenetic relationships. Biodiversity represents the basis of the stability of the ecosystems, providing environmental resilience [14]. Assessing microbial biodiversity also provides various biotechnological resources for food production and regenerative agriculture, environmentally-friendly technologies such as bioremediation and bioprospection of new enzymes, antibiotics, and biological processes. Studies on microbial diversity and taxonomy are crucial to guarantee the environmental and socioeconomic benefits be still unknown to the microbial world. In one attempt to put a monetary value on goods and services provided by ecosystems, the worth of biodiversity was estimated at the outstanding value of US$ 33 trillion per year, close to the GDP (growth domestic product) of the United States and China combined [15]. Other estimates point out to lower or similar values, but undoubtedly, all of them are important [16].

Although many techniques, rules, and concepts are used for prokaryotes in general, in this review we describe how bacterial taxonomy evolved until genomic era and important tools developed to assess bacterial diversity and guide to proper classification. We also present a case study with rhizobia to clarify how the evolution of the taxonomic science impacted this group of bacteria, probably amongst the most important for ecosystems and agricultural sustainability, and improved our knowledge about them.

## 2. The Bacteria Species Concept and Description of New Taxa

The species concept is considered a universal theory limiting the category “species” for all living organisms. Concerning the prokaryotes, several incongruences are discussed, since they do not fit into the most common eukaryotic species perceptions, such as the morphological, biological, or evolutionary concepts [17]. For years, the bacterial species were classified based on phenotypic features, followed by polyphasic analyses involving phenotypic, genotypic, and phylogenetic properties, but with the methods differing among bacterial groups. As a result, each described taxon is represented by a lower taxon, and a type species represents each genus. Nowadays, the advancement of next-generation sequencing (NGS) has encouraged the description of novel species based mainly on genomic sequences [18], and this practice is revolutionizing the bacterial species concept and the taxonomic groups [19–23].

For the description of novel bacterial taxa, the taxonomists follow guidelines from the International Committee on Systematic of Prokaryotes (ICSP), split into several subcommittees, according to the knowledge areas. Regarding the rhizobial species, the Subcommittee on Taxonomy of Rhizobia and Agrobacteria is responsible for the guidelines. For many years, the only guideline for rhizobia taxa description available was published by Graham and collaborators in 1991 [24]. More recently, subcommittee members proposed an improvement to the minimal standards for the species and genera description [25], provided on the website https://sites.google.com/view/taxonomyagrorhizo/home. The genomic profile comparison of strains studied and related type strains is required among the updates, and the genome sequence of the type strain representing the new species must be deposited in databases. This requirement will increase the number of genomes available for further studies.

Another critical step in taxonomy concerns the proper nomenclature, which the ICNP regulates. The scientific name of a novel bacterial taxon needs to be in Latin, referring to the history of the taxon, and be published in the “Approved List” of prokaryotes to become a valid name. In the International Journal of Systematic and Evolutionary Microbiology (IJSEM), the official journal of the ICSP, a clear statement of the name (i.e., fam. nov., gen. nov., sp. nov., etc.) and its etymology, as well as the characterization data of the taxon and the type strain designation, must be provided [26]. The valid names outside the IJSEM must follow these same guidelines and request the official journal validation [27].

The List of Prokaryotic names with Standing in Nomenclature (LPSN) is an online tool constantly updated by the Leibniz Institute DSMZ—German Collection of Microorganisms and Cell Cultures GmbH (https://lpsn.dsmz.de/). It includes a broad range of taxonomic information for each described taxon, such as etymology, nomenclatural and taxonomic status, type strain designations, and the link to the description publication [13]. However, it is worth mentioning that some prokaryotes, such as those still uncultivable, or genetic material retrieved from the environment, although might have sequences indicating that could represent new species, do not have sufficient information required to be described as a novel taxon [28]. Therefore, these organisms are classified as a “candidate” of a new species or a new genus, receiving the taxonomic status *Candidatus* [29]. Nowadays, with the development of metagenomics, which involves acquiring genome sequences from sampled materials of a community of organisms inhabiting a common environment, millions of 16S rRNA sequences have been deposited in databases [30], and a large number of them share less than the suggested threshold value of similarity for species circumscription. Therefore, even though the uncultivable prokaryotes have not been isolated yet, the analyses of those sequences imply that they might represent different species. Given this evidence, many efforts have been carried out for gene sequences of uncultivable taxa to be considered by ICSP and to change the status of *Candidatus* to valid names [31].

## 3. A Brief View of Bacteria Taxonomy

Ferdinand Cohn developed the first studies of bacterial classification in the 1870s. He used morphologic traits as criteria to define six different genera [1]. From this, different methods of classification based on phenotypic traits were published, making the studies of taxonomists difficult because of the lack of minimal standards and common agreement on classification [17]. From the first edition of the Bergey's Manual of Determinative Bacteriology (1984–1989), this status was changed, with the definition of a reference guideline for bacterial classification, providing conditions for the microbiologists to merge the criteria adopted among them [32].

Even though the clustering methods emerged before the advent of computers, they were applied in bacteriology just after using computers, around 1957, by Sneath and Ludwig et al. [33, 34]. This era of bacterial taxonomy was marked by numerical studies where several phenotypic properties were electronically tabulated and used as a relatedness measurement. However, although the numerical taxonomy improved the identification of bacteria, it did not consider phylogenetic analyses [35]. Together with the evolution of numerical taxonomy, new techniques considering the physiologic and biochemical traits started to be developed, such as chromatographic and electrophoretic methods to define chemotaxonomic markers, aiming to reach the goal of a universal classification system for bacteria [17].

In the early 1960s, the development of molecular techniques supported the inclusion of methodologies such as the DNA guanine and cytosine content (GC mol%) and DNA-DNA hybridization (DDH) [36, 37] for taxonomic studies. Those techniques allowed the comparison of genomes, improving the classification of bacteria. Since the first experiments based on single-stranded DNA reassociation conducted by Schildkraut et al. in 1961 [38], and for about 50 years, the DDH was used as a standard technique for bacterial species circumscription [8, 9, 17].

A remarkable breakthrough in the attempts to determine relationships between distantly related organisms came around the 1970s when the Taq polymerase enzyme was discovered and used for DNA amplification through the polymerase chain reaction (PCR) techniques [39], and the dideoxy sequencing technology was described by Sanger et al. [40]. Almost simultaneously, the small ribosomal subunits 16S rRNA for prokaryotes and 18S rRNA for eukaryotes were described and started to be broadly used as molecular markers to organize all living organisms into three superior taxa, Archaea, Bacteria, and Eukarya, named as domains [41]. This new hierarchical taxonomic system based on these molecular markers allowed the incorporation of phylogenetic information in the prokaryotic classification [2, 42–45].

The phylogeny studies the evolutionary relationships among organisms, and using conserved molecular data became commonly accepted in taxonomy. After the ribosomal sequences, other conserved genes started to be used [46]. Although less sequences can be used, the Multilocus Sequence Typing (MLST) generally evaluates 8 to 12 concatenated housekeeping genes or other protein-coding genes to identify genotypes and differentiate closely related strains, becoming broadly applied in molecular epidemiology. In the MLST, each gene (locus) contains different alleles; the allelic differences are converted in values resulting in the “sequence type” for the bacteria, which are available in a specific database for comparisons (as https://pubmlst.org/) of several pathogenic bacteria. Even though the MLST is not commonly used to infer phylogeny in epidemiology studies, it was applied for this purpose in taxonomic studies, contributing to the development of the Multilocus Sequence Analysis (MLSA) [47–52].

The MLSA accesses the evolutionary information of concatenated housekeeping genes to build phylogenetic trees with more robust data than the analysis with single sequences [53, 54]. It is an important tool for studying prokaryotic diversity and classifying taxonomic groups due to its high-resolution power on species delineation. The sequences are aligned, and informative sites are compared. As in the 16S rRNA analysis, the nucleotide identity (NI), which is a percentage of sequence similarity, must respect the threshold values suggested for each respective group of study [46, 55, 56]. The phylogenetic reconstructions can be achieved using different classes of inference methods. Each one has its particular strengths and weaknesses, requiring careful considerations to choose the best method that fits the analysis [25, 57].

Today, advanced sequencing technologies allow the taxonomy to use genomic data in silico to compare microorganisms, helping to allocate them in their respective taxa or describe new taxa to accommodate the new group. With sequenced genomes, the taxonomists can calculate the overall genome-related indices (OGRIs) and estimate the relatedness among microorganisms; however, suggested threshold values must also be considered. The OGRIs came to replace the DDH due to its low cost, reproducibility, and quality of genomic information. Furthermore, the genome sequences can be deposited in databases so that other scientists can use the data without cultivating the respective bacteria [51, 58]. The OGRIs include average nucleotide identity (ANI) and digital DNA-DNA hybridization (dDDH), the most broadly used to calculate the relatedness between orthological sequences of two genomes. Those sequences are descended from the same ancestral sequence, which kept conserved across the evolution. Studies showing that ANI is a robust measure of evolutionary distance that correlates with DDH values have been published since 2005 [59, 60].

In conclusion, until the genomic era, the polyphasic taxonomy was used to identify, classify, and name prokaryotes according to phenotypic, genotypic, and phylogenetic characteristics. It enabled considerable progress and stability in microbial taxonomy. However, with advancements in genome sequencing, there are today better tools to delineate species, study phylogeny, and ordinate microbial diversity. The history of microbial taxonomy incorporates the most advanced technologies and adheres to standards and rules, representing a scientific field where the progress goes alongside conservatism [61, 62].

## 4. Bioinformatics, Evolutionary Markers, and Threshold Values

The evolutionary molecular markers are constitutive genes that reflect the phylogeny of the organisms because the bases' substitution on DNA sequences (given by mutations and recombination) is proportional to the evolution that each species underwent from its ancestors, allowing estimates of the differentiation level of the species [63]. In bacterial taxonomy, those molecular markers started to be amplified, sequenced, and used to phylogenetically ordinate bacteria with the development of the PCR and sequencing techniques. The sequences from amplicons of molecular markers of strains under study and related strains need to be aligned using specific algorithms, such as MUSCLE [64] and ClustalW [65], to identify significant sites for the analysis.

In the next step, it is recommended to choose the best substitution model for the multiple sequence alignment, which depends on the phylogenetic method used to understand the phylogeny of the group under study. Models of substitution are algorithms responsible for evaluating the frequency of each nucleotide and its frequency of substitution, differentiating between transitions (exchange for nitrogen bases of the same biochemical class) and transversions (exchange for nitrogen bases of a different biochemical class). With this, the models can infer the evolutive history for the alignment [66, 67]. Finally, the phylogenetic methods are used to construct the phylogenies based on the alignment and best evolutive model. The result is a graphic representation of the evolution of the strains given by a phylogenetic tree.

In a phylogenetic tree, the extremities are represented by the investigated lineages (sequences, strains). The horizontal lines are called branches, and the nodes that connect the branches represent the most recent common ancestry among the strains [67]. However, a reliable phylogeny reconstruction needs to consider the number of sequences, the size of the alignment, the statistical support, and the outgroup. The bootstrap is commonly used in phylogenies to provide statistical support for the tree nodes [68]. This confidence test consists of multiple resampling of a dataset with the same size as the original alignment. For each resampling, the algorithm randomly chooses the sites, that is, the columns of the alignment, which may be repeated or excluded. Different resamplings result in phylogenetic trees that are compared with the tree obtained in the original alignment. The number of resamplings is variable and may be chosen by the researcher to ensure a reliable phylogeny [69]. The bootstrap value corresponds to the percentage of each group found among all trees obtained in the resampling. The outgroup represents a related taxonomic group, and it is used to show the ancestral strains, helping to determine the tree's root [70].

It is also possible to calculate the NI, a mathematic parameter used to measure the percentage of similarity among the nucleotide sequences from the alignment, but it does not include evolutionary analyses. Specific software is available to calculate the NI, such as BioEdit [71]. Several studies compare NI of particular sequences and genomic analysis, such as dDDH or ANI, of a large group of strains to suggest standard values of NI that reflect whole-genome features of prokaryotes, saving time and costs. These values are commonly known as threshold or cutoff values and are used for taxon circumscription [56, 72].

The 16S rRNA gene, a critical molecular marker in bacterial taxonomy, contains approximately 1,500 base pairs (bp) and plays a role in synthesizing essential proteins to the functioning of every prokaryote. It is originated from a common ancestor among all prokaryotes, being homologous and keeping conserved throughout the evolution process, but having variable sites with evolutionary information [42, 73]. The degree of divergence in the 16S rRNA sequences allows to estimate the phylogenetic distance among strains and, for this reason, is considered a molecular chronometer in bacterial taxonomy [1, 67].

Some numerical values of 16S rRNA NI have been suggested to delimit species boundaries. For example, Stackebrandt and Goebel, in 1994 [74] suggested that strains sharing less than 97% NI on the 16S rRNA sequences should not belong to the same species because the homology would not give a DNA-DNA reassociation above 60–70%. After that, a threshold ranging from 98.7 to 99% was recognized as corresponding to the DDH reassociation value for species delineation [1]. More recently, Kim et al. [72] carried out a large comparative study between 16S rRNA sequence similarities and ANI and proposed the threshold of 98.65% similarity in the 16S rRNA sequence for differentiating two species.

The increase in the number of novel taxa described using the 16S rRNA sequence data has revolutionized our knowledge of the microbial taxonomy, especially at the species level [51]. However, the resolution power of the 16S rRNA analysis is limited due to the predominance of conserved sites in these sequences, not giving enough evolutionary information for the analysis and restricting the identification at the genus level [6, 75–77], with the suggested threshold of 95% for genus delimitation [78]. In addition, studies have reported that those sequences can have multiple heterogeneous copies and, in some groups of microorganisms, the horizontal gene transference (HGT) may happen [79, 80]. These events may lead to misinterpretation of the evolutionary data [81]. Concerning these issues involving the ribosomal sequences, the information from the 16S rRNA phylogeny must corroborate with the suggested values of molecular analyses with a better resolution power and should not replace other methodologies.

Many taxonomists analyzed the internal transcribed spacer (ITS) as an alternative molecular marker to increase the knowledge about the ribosomal region [75, 82–84]. Those sequences are located between the small-subunit (16S rRNA) and the large subunit (23S rRNA) of the ribosomal RNA and contain highly variable sequence regions, allowing the differentiation of bacteria even at the strain level, showing higher phylogenetic diversity [85–87]. For example, a study with the *Bradyrhizobium* genus demonstrated that strains sharing 95.5% NI on the ITS sequences would correspond to 60% of reassociation on the DDH, belonging to the same species [88]. However, as the 16S rRNA, these sequences do not have a high-resolution power to infer taxonomic groups at the species level. Despite being not often used, the 23S rRNA may also be applied as a molecular marker. The 23S rRNA is located in the large subunit of the prokaryotic ribosome, has a conserved function, universal distribution, and presents sequences with different levels of variation. However, it is commonly characterized by showing insertions and deletions with large sizes, resulting in more information to improve phylogenetic resolution as an additional analysis [66, 89].

Even though the 16S rRNA sequences have been broadly used as an effective tool for basic evolutionary analyses of cultivable and uncultivable bacteria, for closely related groups, they are unable to determine their nearest neighbors since different species can share identical or nearly identical 16S rRNA sequences [83, 90–93]. An approach that can be used to fill this gap is the MLSA. As mentioned before, the MLSA puts together evolutionary information from concatenated housekeeping genes to better assure the correct taxa identification of strains under study. The housekeeping genes are responsible for fundamental roles in the basal microbial metabolism, encoding for essential proteins of the cell functions. Therefore, they have high conservation levels but faster evolutionary rates when compared with the ribosomal sequences, carrying more phylogenetic information [94].

The MLSA allows the analysis of genes together as a larger phylogenetic dataset [35, 46, 52, 95]. Therefore, the methodology aggregates sufficient phylogenetic signals, offering a buffer effect to the noncongruent signals that influence smaller datasets, being more reliable [77, 94, 96]. However, it is worth mentioning that the single housekeeping genes analysis does not reflect the entire evolutionary pattern of the strain; it is recommended to proceed to the comparison between the 16S rRNA and each housekeeping gene phylogeny to detect and avoid potential HGT and recombination events, since each housekeeping gene may have a different history of evolution [75, 92].

The main requirements of MLSA involve the selection of housekeeping genes that should be present in the genome of all organisms object of study as a single copy and spread in the genome. They must also have a consistent size to allow phylogenetic reconstructions and sequencing. Consequently, different genera may vary in the set of genes used in the analysis [46, 52]. In addition, the resulting data from MLSA must corroborate with the 16S rRNA, dDDH, and ANI analyses. It is recommended to use at least five housekeeping genes, considering that the number of genes reflects directly in the discriminatory power of the technique [52]. Some of the most common housekeeping genes applied for the classification of new taxa are ATP synthase *β*-subunit (*atpD*), chaperone protein (*dnaK*), glutamine synthase II (*glnII*), glutamate synthase (*gltB*), DNA gyrase *β*-subunit (*gyrB*), recombinase A (*recA*), RNA polymerase *β*-subunit (*rpoB*), and tryptophan synthase *β*-subunit (*trpB*) [10, 75, 97–99]. In addition, new genes can be studied, aiming to determine if they provide relevant and congruent phylogenetic information as those genes cited above.

After the housekeeping genes selection, each set of single-gene sequences should be aligned and trimmed to keep the same region of comparison and the same size for the alignment. Subsequently, the phylogeny of each single gene is individually built and compared to each other, and if they are congruent, the alignments must be concatenated to proceed with the MLSA. The concatenation process can be carried out manually using software or any text editor program. Some of the most common software for alignment of prokaryotic sequences are MEGA (Molecular Evolutionary Genetics Analysis) [100] which provides alignment tools as MUSCLE and ClustalW, and BioEdit [71] which also provides the alignment tool ClustalW. To concatenate the sequences, the SeaView [101] is commonly used. In addition, it is important to search for updated sequences in databases to construct reliable phylogenies, verifying the quality of the sequences and the availability of the sequences of the closely related strains.

In 2002, the ad hoc committee of the ICSP recommended the analysis of the concatenated housekeeping genes as a promising method to replace the DDH association in bacterial taxonomy [102]. Following this, Konstantinidis et al. [56] studied extensive whole-genome comparisons of four important bacterial groups. They concluded that the phylogeny of six to eight genes reflects the threshold of 70% DDH and 96% ANI for species circumscription. The authors did not suggest any specific set of genes and affirmed that even if the genes are randomly combined, they provide a robust phylogeny for the group studied [56, 103]. Since then, the NI of concatenated housekeeping genes has been accepted to predict the whole-genome information and differentiate bacterial groups [98, 104–106]. However, no universal threshold for MLSA has been determined for species circumscription yet [18]. Nevertheless, the MLSA has been used as a great strategy to differentiate species in bacterial taxonomic studies, and it is considered as a critical step of bacterial taxonomy since it is more phylogenetic sensible than 16S rRNA analysis and involves conserved regions able to infer phylogeny, unlike OGRIs, which are mathematic parameters [51].

## 5. Evaluating Genomic Traits

As the DNA molecules represent the identity of the species, studying the genomic profiles allows obtaining relevant information for taxonomic purposes. Prokaryotic genomes contain repetitive sequences distributed throughout the chromosome; however, the sites, length, and the number of times they are repeated are characteristic of each strain, representing a fingerprint of each genome. To evaluate those genomic profiles, the taxonomists use DNA amplification by PCR with specific primers for those regions or restriction enzymes to cut the chromosome in the restriction sites. As a result, in both procedures, there is a mixture of different fragments of DNA that can be separated by electrophoresis revealing the respective genetic profile [9, 107, 108]. For taxonomic studies, there are three main sets of repetitive elements: repetitive extragenic palindromic (REP) [109], enterobacterial repetitive intergenic consensus (ERIC) [110], and BOX elements [111–113]. Those analyses are limited for species circumscription, but if properly used, they can be applied to identify variability from strains of the same species, to find clones, and to authenticate strains in a study or culture collection [8, 108, 114].

The DDH evaluates the extension and stability of DNA hybrid strands after the dissociation and consecutive reassociation of a two-genomes mixture incubated under controlled conditions [115]. The cutoff is based on the percentage of reassociation using the difference in melting temperature of the hybrid DNA strand (heterologous) compared to 100% of the original strand (homologous). If this association is 70% or higher, or the melting temperature difference (ΔTm) is below 5°C, the genomes compared belong to the same species [7, 18, 35]. The DDH started to be applied when a putative novel group shared more than 97% NI in the 16S rRNA sequence with the related species, aiming to assure enough difference among the genomes of the strains to support the description of a new group. Since the introduction of the DDH in microbiology [116], it became the “gold standard” for species delineation of Bacteria and Archaea and a required technique in every prokaryote species description [8]. However, despite the cited advantages, the DDH has limitations, especially for being time-consuming, requiring intensive labor, and not allowing comparison of results obtained in different experiments, not enabling establishing a global database [44, 51, 117, 118].

The first prokaryote genome sequenced was of the bacteria *Haemophilus influenzae* in 1995, using the conventional Sanger sequencing technique [119]. Although a new genomic era was starting, the high cost and time-consuming process of genome sequencing did not allow significant progress for one decade. In 2005, with the arrival of the NGS technologies, also known as high-throughput sequencing methods, the easy access and low cost of the genome sequencing launched the report of sequenced genomes of prokaryotes [51, 120]. Today, many sequencing platforms can be used, and they are split into two main groups, depending on the type of template used for the sequencing reactions, the high-end instruments, and the bench-top instruments. The high-end platforms demand more technology infrastructure for data tracking, analysis, and storage, while the bench-top ones have more modest requirements. Nowadays, the most popular platforms are the MiSeq and HiSeq (Illumina, USA), Ion Torrent (Thermo Fisher Scientific, USA), and Pacific Biosciences (USA) [60].

The statistical parameters used to report the quality of the genome assembly recommended for taxonomic purposes are (i) the genome size, defined as the length of all contigs sequenced; (ii) the N50, defined as the length of the shortest contig that accumulatively shows 50% or more of the genome size when the lengths of the contigs are summed from the largest to shortest; and (iii) the depth of coverage from the sequencing, indicating how many sequencing reads are generated; this value is usually given as folds and is recommended a minimum of 50X (50-fold) for the platforms cited before [60, 121]. Another critical issue to consider in taxonomy is the genome authenticity, which can be achieved by comparing complete conserved sequences, as the 16S rRNA or housekeeping genes extracted from the genome, with the same sequence obtained through the conventional Sanger sequencing. When describing a new species, this comparison should be performed at least with the type strain [51].

Presently, using genome sequencing, the taxonomists can use other analyses to study the relatedness among the DNAs of bacteria. The threshold values suggested for these analyses relate to the 70% from the DDH technique. As mentioned above, these values are known as OGRIs and effectively calculate genomes' similarities *in silico* [51, 58]. The ANI and the dDDH are now extensively used to replace the original DDH, adopting the threshold values of 95–96% and 70%, respectively, for species circumscription. The OGRIs can be calculated using software tools for taxonomic purposes. Today there are many readily available web services, such as the Genome-to-Genome Distance Calculator and the ANI calculator, and standalone tools, such as JSpecies and OrthoANI with USEARCH. Besides being fast and of low cost, the high quality of the sequences allows the improvement of genomic databases that researchers can use worldwide [60, 117, 122, 123].

With the availability of genome sequences, another parameter that can be calculated *in silico* is the DNA guanine and cytosine (GC) content, which is also used as a genotypic marker in taxonomy [124]. In the beginning, the bases' composition variation from strains of the same species should not exceed 3 mol%, and for strains from the same genus, 10 mol% [7]. Later, a survey comparing the GC content with the genomic data and dDDH indicated that the variation should not exceed 1 mol% among strains of the same species [58]. The GC content can be calculated using tools to assemble the genomes, such as the SEED viewer provided by the RAST server [125], QUAST [121], and BioEdit [71]. Although the GC content is important to distinguish nonrelated bacteria, similar DNA base composition does not necessarily imply that the two strains are closely related [35].

## 6. Phenotypic Traits

In contrast to the taxonomy of eukaryotes, where the phenotypic characteristics can be used to differentiate some organisms, these traits are questionable in prokaryotes since different bacterial species can present identical phenotypes [17]. Nevertheless, starting the prokaryotic classification using the phenotypic evaluation may help delimit further analyses; for example, most pathogenic bacterial groups present well-established phenotypes in the literature and are critical to quick diagnostics.

The classical phenotypic tests in microbiology include morphological, physiologic, and biochemical analysis. Morphological characteristics describe the cellular and colony features, such as the cell shape, endospore formation, presence of flagella, Gram stain, color colony, diameter, opacity, mucus production, and their consistency. On the other hand, the physiological and biochemical characteristics include data about the culture under different growth conditions, such as range of temperature, pH (4–12), salinity tolerance (1–10%), O_2_ or CO_2_ requirements, tolerance to different antibiotics, enzymatic activity (i.e., urease), and metabolism of compounds (i.e., carbon source and nitrogen source) [7, 9, 28, 126]. Therefore, it is recommended to compare the data obtained with reference or type strains and to include negative and positive controls [8]. In addition, the use of commercial tests with minimal standards such as API (bioMérieux) or Biolog (Biolog, Inc.) that evaluate the carbon source utilization by bacteria is suggested to avoid incongruences among laboratories.

Another common phenotypic test is the chemical characterization of cells, which evaluates extracellular elements (peptidoglycan, teichoic acids, and mycolic acids), cell membrane composition (fatty acids, polar lipids, respiratory lipoquinones, and pigments), or cytoplasm compounds (polyamines) [8]. These features are also known as chemotaxonomic markers because they are usually stable within a bacterial group [127]. The resulting data are available in specific databases. One of the most common is the database of fatty acids profiles implemented by Sherlock Microbial Identification System (MIDI, Inc.) [8, 105, 128]. Even though the chemical characterization provides important information about the cells, it has become unusual in taxonomic studies because the tests are laborious, require specific equipment and methodologies, and usually allow the classification only at the genus level identification. Lately, the use of Matrix-Assisted Laser Desorption/Ionization Time-of-Flight (MALDI-TOF) for bacterial identification has become a rapid, precise, and cost-effective method, especially compared to traditional phenotypic and molecular techniques. It is based on the analyses of bacteria components profiles, as proteins directly extracted from intact bacteria, and compared with a reference database for identification [129, 130]. This methodology is commonly used on clinical isolates' identification, but as the reference databases are being updated, it is becoming practicable for other groups of bacteria, such as the rhizobia [131–133].

It is worth mentioning that there are many genes coding proteins without known function. Therefore, the phenotypic tests could help the search for proteins with biotechnological interest, improving the knowledge about the interactions of microorganisms with the environment [18]. However, the phenotype results from the combination of genotype and environmental conditions. Consequently, it is impossible to know the whole phenotype of a prokaryote based only on observable characteristics or on the information obtained from the genome. In taxonomy, it is common to find some incongruent data in phenotypic evaluations, probably because the accessory genome codes many phenotypic traits; for example, the plasmids easily exchanged among the organisms. In addition, as there are no laboratory conditions able to mimic the environment entirely, the phenotypic characterization can exclude some microorganisms, such as the uncultivable. Therefore, these data need to be validated by genomic analysis [134].

## 7. Rhizobia Study Case

### 7.1. History of Rhizobia Taxonomy

More than 2,000 years ago, ancient Chinese literature reported that crop rotation of legumes with cereals was traditionally used to enhance grain production. Improvement in soil fertility by cultivating legumes was thus already noticed at that time, although the mechanisms involved were not known yet [28]. However, it was just by the end of the nineteenth century that the assimilation of atmospheric nitrogen was related to the root nodules in legumes, and in 1888 the first root-nodule bacterium was isolated from nodules of *Pisum sativum* plants (pea) and reported by the Dutch microbiologist Martinus Willem Beijerinck to be responsible for the nitrogen fixation process. The isolated bacterium was first named *Bacillus radicicola* but later reclassified as *Rhizobium*, comprising the *Rhizobium leguminosarum* species [28, 135, 136]. Since then, the nodulating nitrogen-fixing bacteria have been generally called rhizobia [137].

In the early twentieth century, nodulation tests using a broad range of host plants and different bacteria were conducted, and the specificity between the host plants and the symbiotic bacteria was reported. Based on this, Baldwin and Fred [138] proposed the cross-nodulation concept, indicating specificity between rhizobia and host plants. For about 80 years, taxonomists used this concept for species definition, and six main species were described: *R. leguminosarum* [136], *R. phaseoli*, *R. trifolii*, and *R. meliloti* [139], which produced acid reaction on yeast-extract mannitol agar (YMA) medium, and *R. lupini* [140] and *R. japonicum* [141], which produced alkaline reaction on the same medium. Besides those six species, some other strains isolated from cowpea were defined as *Rhizobium* spp. [135, 142].

The taxonomy dropped the cross-nodulation concept after several studies reporting both exceptions and strains sharing high similarity and belonging to different groups. Additionally, the rhizobia classification needed to include more information to adjust to the general bacterial taxonomy [143–145]. However, even after the cross-inoculation concept was dropped in rhizobia taxonomy, it continued to be studied, representing an important feature regarding the inoculation and efficiency of the symbiosis [28].

The next step of rhizobia taxonomy was based on the numerical taxonomy using computers to compare bacteria properties. Around the 1960s, many analyses were included in taxonomic studies involving phenotypic traits, growth conditions, nutrient resources, metabolic features, and resistance to antibiotics and other chemicals, among others. Also, the DNA molecule started to be investigated, and the base composition (GC mol%) was added to bacterial classification [145–148]. Using the numerical taxonomy combined with DNA analyses, three other rhizobial genera were described. In 1982, Jordan [149] proposed the description of the genus *Bradyrhizobium* to allocate the slow-growing species *B. japonicum* and *B. lupini*. Six years later, the genus *Azorhizobium* [150] was described, encompassing strains that can effectively nodulate roots and stems of *Sesbania rostrata* and fix nitrogen under free-living aerobic conditions. In the same year, the *Sinorhizobium* genus was proposed for the fast-growing soybean species *Rhizobium fredii* [151].

Around the 1990s, many other analyses were included in taxonomic studies. The polyphasic taxonomy confirmed some of the taxa proposed with the numerical taxonomy but also pointed out that the numerical taxonomy lacked information about the evolutionary relationships among rhizobia. Consistent DNA studies allowed the taxonomists to assess the diversity and phylogenetic relationship among bacteria at a molecular level [28]. The 16S rRNA sequence analysis was included in rhizobia species descriptions and reclassifications in 1991 when Graham and collaborators [24] published the minimal standards for species description of new rhizobia and *Agrobacterium* [152]. Considering the phylogeny and similarity of 16S rRNA, rhizobia were classified in the phylum Proteobacteria, subdivision *α*-Proteobacteria [153]. In the same decade, two more genera were described. First, Jarvis et al. [154] proposed the genus *Mesorhizobium* to allocate five *Rhizobium* species with an intermediate growth rate than fast-growing *Rhizobium* and *Sinorhizobium* and slow-growing *Bradyrhizobium*. Following, de Lajudie et al. [155] proposed the description of the genus *Allorhizobium* of symbiotic bacteria associated with the aquatic legume *Neptunia natans*.

The six rhizobia genera were allocated in four distinct families: *Bradyrhizobium* [149], *Mesorhizobium* [154], and *Azorhizobium* [150] belonging to families Bradyrhizobiaceae (today Nitrobacteraceae), Phyllobacteriaceae, and Xanthobacteraceae, respectively, and *Rhizobium* [136], *Sinorhizobium* (reclassified as *Ensifer*) [151, 156], and *Allorhizobium* [155] belonging to the family Rhizobiaceae [157]. Additionally, a study with members of the family Rhizobiaceae reported similarities of the 16S rRNA gene higher than 92% among the genera, suggesting that this value could be a threshold for family delineation [158].

The new century started a revolution on the rhizobia taxonomy. A first milestone occurred in January of 2001, with the first report of a non-rhizobia nitrogen-fixing legume-symbiotic bacterium isolated from the nodules of *Crotalaria*, classified as *Methylobacterium* [159], with the species *M. nodulans* [160]. In the same month, based on the 16S rRNA analysis [161], Young et al. [162] suggested the inclusion of all species of *Agrobacterium* [152] and *Allorhizobium undicola* [155] in the genus *Rhizobium.* In 2002, a new species of the genus *Devosia* was reported to induce nitrogen-fixing root-nodules in *Neptunia natans* [163]. In 2005, the first rhizobia in the genus *Phyllobacterium* were described as *P. trifolii*, isolated from the nodules of *Trifolium pratense* [164]. From 2005 to 2007, the genus *Ochrobactrum* (today, *Brucella*) [165, 166] allocated two nodulating species, *O. lupini* and *O. cytisi* [167, 168], isolated from nodules of *Lupinus albus* and *Cytisus scoparius*, respectively. In 2008, Lin and collaborators [169] described in the *Shinella* genus [170] the symbiotic bacterium *S. kummerowiae* isolated from root nodules of *Kummerowia stipulacea.* In 2009, the first rhizobial isolate (BA135) belonging to the species *Aminobacter aminovorans* was reported, isolated from nodules of *Lotus tenuis* [171]. In the following decade, from 2012 to 2014, the genus *Microvirga* [172] allocated four nodulating and nitrogen-fixing new species, *M. lupini* isolated from *Lupinus texensis*, *M. lotononidis*, and *M. zambiensis* isolated from *Listia angolensis*, and *M. vignae* isolated from *Vigna unguiculata* [173, 174].

Besides all those changes in the *α*-Proteobacteria class, 2001 was outstanding by the report of a nodulating *β*-Proteobacteria [175], belonging to the genus *Burkholderia*, described by Yabuuchi and collaborators in 1992 [176]. It was the first time that the essential nodulation genes (*nod*) and the nodulation capacity were reported in symbiotic bacteria not belonging to *α*-Proteobacteria [175]. After that, several nodulating bacteria of *β*-Proteobacteria genera were described, including the *Ralstonia* genus [177], reclassified in 2004 as *Cupriavidus* [178–180], in addition to several species of the *Burkholderia* genus, later reclassified as the new genus *Paraburkholderia* [181, 182]. In the following years, the introduction of single and concatenated housekeeping genes in phylogenetic studies culminated in the reclassification of many species and the proposal of the new genera *Neorhizobium* and *Pararhizobium*, and also the revision of the genera *Agrobacterium* and *Allorhizobium* [183, 184]. More recently, using whole-genome analyses, Santos and collaborators [21] suggested the description of two new genera, *Mycetohabitans* and *Trinickia*, this last one containing the nodulating nitrogen-fixing species *T. symbiotica*.

With the evolution of taxonomic analyses, we may conclude that many descriptions of nodulating bacteria, isolated from nodules of different hosts and belonging to nonrhizobial genera have been published, and many taxonomic groups were reclassified. Most of those bacteria have a diverse set of nodulation (*nod*) and nitrogen fixation (*nif*) genes, some of which are related to genes of different members of classical rhizobial genera. All those findings show that the ability to establish symbiosis with legumes is more widespread in bacteria than anticipated before [137, 175].

Today, rhizobia are distributed in eight families, seven belonging to the *α*-Proteobacteria class and one in the *β*-Proteobacteria. From both subclasses, the *α*-rhizobia are reported as broadly distributed, both in geography and host-plant range, and although the *β*-rhizobia are well established, their distribution seems more restricted [28]. The *α*-Proteobacteria families are the Rhizobiaceae that allocate seven genera, the Phyllobacteriaceae with three genera, the Methylobacteriaceae with two genera, and the other four families allocating only one genus, Nitrobacteriaceae (old Bradyrhizobiaceae), Brucellaceae, Hyphomicrobiaceae, and Xanthobacteiaceae. The *β*-Proteobacteria family is Burkholderiaceae with three genera. The list of genera and the number of species with valid names standing in nomenclature (without synonyms) according to the LPSN in October of 2021 are listed in Table 1.

In 2020, a study performed phenotypic, genomic, and phylogenetic analyses of the genus *Ensifer* and suggested that the genus should be separated into two genera, one for the symbiotic clade and the other for the nonsymbiotic clade [185]. More recently, a publication on the IJSEM suggested the revision of family Rhizobiaceae [186]. The extensive study suggests a threshold for core-proteome average amino acid identity (cpAAI) of approximately 86% as a new framework for genus delimitation. It is noteworthy to mention that cpAAI must not be used as sole information for genus delimitation, and the authors specify “approximately 86%” to provide some flexibility regarding the evolution of each genus. Although not yet updated in the LPSN website as validated names, among the reclassifications based on the study of Kuzmanović and collaborators [186], there are arguments that the unification of the genera *Ensifer* and *Sinorhizobium* is no longer justified, and eight new combinations were suggested, but not all involving rhizobial strains [186].

From Table 1 based on the LPSN, we may conclude that today over 200 known rhizobial species are split into 19 genera of the *α*- and *β*-Proteobacteria subclasses, and the number increases every year. However, less than half of these valid names from the 19 genera have species comprising strains already reported for their symbiotic properties, including nodulation and nitrogen fixation abilities. Furthermore, many species are reported as endophytes, or were isolated from environmental samples, or from nodules but unable to reestablish symbiotic associations. Therefore, the symbiotic capacity remains largely unknown for many species.

Additionally, in 2004, Benhizia and collaborators [187] published for the first time the isolation of *γ*-Proteobacteria species from legume nodules. In this study, 52 isolates belonging to the *Pseudomonas*, *Escherichia*, *Leclercia*, *Pantoea*, and *Enterobacter* genera were isolated from three *Hedysarum* species, and rhizobia-like bacteria were found occupying the nodules. However, Koch's postulates and the symbiotic parameters from the isolates were not investigated. Shiraishi and collaborators [188] also reported in 2010 the existence of rhizobial strains in the *γ*-Proteobacteria subdivision. The authors described *nod* and *nif* genes in the *Pseudomonas* sp. strain Ch10048, sharing high similarity with the symbiotic sequences of *Agrobacterium* sp., suggesting the acquisition of these genes through HGT from rhizobial species in the soil. Despite the report of *nod* and *nif* genes in strain Ch10048, and the confirmation of the ability to nodulate the host legume *Robinia pseudoacacia*, the existence of *γ*-rhizobia remains controversial until additional evidence confirms that the genes were not provided by other bacteria coexisting in the nodules and that the nitrogen fixation ability of the strain is tested [28].

Undoubtedly, rhizobia taxonomy advanced together with prokaryotes' taxonomy, and improvements regarding the origin and evolution of these bacteria were obtained. However, there is a need to increase the studies relating taxonomy and phylogeny with the phylogeny of nitrogen fixation and biotechnological properties of rhizobia.

## 8. Rhizobial Symbiotic Parameters and Genome Architecture

As commented before, the members of the Subcommittee on Taxonomy of Rhizobia and Agrobacteria of the ICSP reviewed the taxonomic developments for this group of bacteria and updated the minimal standards for taxonomic studies, including additional considerations specific to rhizobia and agrobacteria. According to them, taxonomic definitions should not include symbiotic or pathogenic characters because the interactions with plants are determined by accessory genes that may be present in several bacterial species, and be gained or lost, imposing taxonomic limits [25]. Instead, if a strain has a phenotype regarding plant-interaction, this should be described in the taxonomic proposal but considered as a property of the strain, not of the whole taxon. It is worth mentioning that symbiovars are also studied in rhizobial surveys, although not accepted as a formal taxonomic category. The term symbiovar was proposed in 2011 to name a group of strains able to nodulate and fix nitrogen with a range of legumes or a specific legume, and today this definition is based mainly on symbiotic genes' phylogeny [25, 189].

Concerning the description of new rhizobial species, it is especially recommended to evaluate the symbiotic ability of the strains based on Koch's postulates using the original host and/or other legume species. This last alternative may be used to expand the information about the host range of the strains and to define symbiovar groups or when the seeds of the original host plant are not available. The species *Phaseolus vulgaris*, *Macroptilium atropurpureum*, *Vigna unguiculata*, and *Mimosa pudica* are promiscuous legumes commonly used in taxonomic studies of rhizobia. The symbiotic ability may be evaluated compared to negative controls by the presence/absence of root nodules, plant biomass, N content, or the acetylene reduction assay. In addition, the strains must be reisolated from the nodules, keeping the original phenotypic, phylogenetic, or genotypic features, obeying Koch's postulates [25, 190, 191].

Based on the meta-analysis of 1,708 completed bacterial genomes performed in 2017 by diCenzo and Finan [192], the average and median of bacterial genomes found were 3.65 Mb and 3.46 Mb, respectively. In a review study of 2020, Geddes and collaborators [193] compared the genomes of representative strains of *α*-rhizobia and *β*-rhizobia and showed that rhizobial genomes range from 3.42 Mb in *Cupriavidus taiwanensis* LMG 19424 to 9.36 Mb in *Microvirga lupini* Lut6. However, the authors highlighted that some strains of *Bradyrhizobium, Mesorhizobium*, and *Azorhizobium* genera might have higher genomes, which means that the rhizobial genomes can be twice or more times higher than the average size of bacterial genomes reported in the two studies [192, 193]. In contrast, another study reported that *Ensifer* strains from the symbiotic clade carried an average of 325 fewer genes and appeared to have fewer rRNA operons when compared to strains belonging to the nonsymbiotic clade [185]. Large genomes may be related to adaptations to the soil and rhizosphere conditions [194].

In general, the rhizobial genes responsible for plant infection, nodulation, and nitrogen fixation are clustered together in symbiotic plasmids or symbiotic islands in the chromosome, or even in both genomic regions [195–200]. Those clusters are frequently part of mobile genetic elements (MGE) that have independent evolutionary pathways [201]. An exception was first reported in 2007 revealing a group of Bradyrhizobium strains with photosynthetic ability that does not possess nodulation genes and can induce nodulation without nodulation (Nod) factors [202]. It is known that Nod factors play a crucial role in host specificity in the rhizobia-legume interactions; those molecules differ on the symbiosis specific backbone length and other structures, determining the set of plants that the rhizobia can nodulate [203, 204]. In a review recently published, Patra and Mandal [205] pointed out other studies reporting that even in absence of Nod factors, other bradyrhizobia strains, not belonging to the photosynthetic group, are also able to establish successful nodulation. Among the hypotheses discussed, there is the possibility that Nod factors independent nodulation start with the host infection through crack invasion process, instead of the formation of the common infection thread. After the infection, the nodulation might take place using similar signals and mechanisms present in Nod-dependent nodulation [205, 206].

Besides the main chromosome, some bacteria have a “second chromosome” or “megaplasmid,” for which the term “chromid” was proposed. These elements have some core genes and nucleotide composition similar to the associated chromosomes, but most of their genes are accessory. Some rhizobia and agrobacteria also have genus-specific chromids, similar within a genus but with different sets of conserved genes among genera. An example is some *Agrobacterium* species with linear chromids carrying a unique replication system and conserved genes [207].

## 9. Rhizobial Origin Hypothesis and Evolution

As biological nitrogen fixation is considered one of the most important biological processes for life on Earth, there is great biotechnological interest in diazotrophic bacteria [208]. Studying the nodulation ability and nitrogen fixation efficiency, together with the phylogenetic comparison of core and symbiotic genes, gives insights about the origin of the diazotrophic bacteria and the evolution of the biological fixation ability on prokaryotes [106, 209, 210].

Remigi and collaborators [201] reviewed that the nitrogen fixation ability is older than the nodulation process and broadly spread among Bacteria and Archaea. The nodulation genes might have clustered early in the symbiosis evolution path by duplication and specialization of other functional genes. Nowadays, the high diversity of nodulation genes makes it difficult to suggest which bacterial lineage was ancestral. The *nif* and *nod* genes have different phylogenies, implying that rhizobia inherited the nitrogen fixation ability of their free-living relatives [211]. Besides, as the proximity of those genes is not essential for function, it suggests a relatively recent HGT event as a symbiosis set.

Evidence indicates that the *Bradyrhizobium* genus might be the rhizobia's ancestor [212]. Using the genes from the glutamine synthetase enzymes (GSI and GSII), essential for nitrogen assimilation, the estimates are that *Bradyrhizobium* originated 553 million years ago (MYA). Other rhizobia evolved 400-324 MYA, originating the *Mesorhizobium*, *Rhizobium,* and *Sinorhizobium* (=*Ensifer*) genera. Interestingly, the first legumes ascended on Earth long after, around 70 MYA [63, 213]. Another piece of evidence of *Bradyrhizobium* ancestry is that some strains were detected with nitrogen fixation ability as free-living bacteria, as observed in some *Azorhizobium*, and both lineages are very distant from the other rhizobial genera [28].

It is well known that bacteria have different mechanisms to exchange genetic material. This event is more recurrent among organisms sharing the same ecological environment, reinforcing that some rhizobia evolved by acquiring symbiosis genes from other species by HGT [197, 214, 215]. Furthermore, a study with *A. caulinodans* reported an increase in horizontal transference frequency of its symbiosis island in the legume rhizosphere or in the presence of plant flavonoids, suggesting a host-dependent evolution [216]. Over evolutionary time, the horizontal transference of symbiotic functional genes among symbiotic and nonsymbiotic bacteria is hypothetically responsible for the growing number of studies reporting great rhizobia diversity [201, 217]. Given the vast population of rhizospheric bacteria, it might seem paradoxical that the symbiosis is restricted to only nineteen genera. However, an increasing number of studies report the coexistence of nonrhizobial bacteria inside the nodules, which deserve further studies and might help to explain that additional partners help the symbiosis [153, 218–222].

## 10. Conclusions

**Figure 1:**
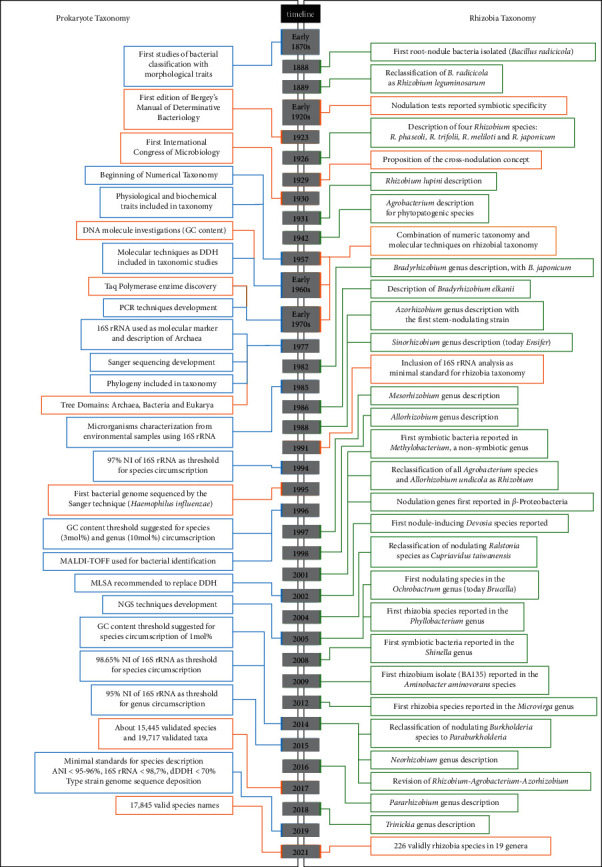
Timeline showing the evolution of methods and achievements in prokaryote and rhizobia taxonomy.

As presented in this review, the main goal of taxonomy is to ordinate living organisms in a stable and hierarchical system. As shown in Figure 1, remarkable progress has been achieved in both prokaryote and rhizobia taxonomy and phylogeny. However, profound changes may arise with the genomic era. Nevertheless, robust taxonomic methodologies are becoming gradually available in an increasing number of laboratories, allowing researchers to conduct surveys of great interest. These studies have contributed to new insights about the origin, evolution, and diversity of bacteria on Earth and the description of almost 18,000 valid species, of which more than 220 are rhizobia. However, more studies are needed to correlate taxonomy with biotechnological properties of nitrogen-fixing rhizobia to improve their contribution to agricultural and environmental sustainability.

## Figures and Tables

**Table 1:** List of genera comprising rhizobial species with valid names standing in nomenclature (LPSN) in October 2021. The number of valid names does not include synonyms.

*α*-Proteobacteria		
*Hyphomicrobiales (=Rhizobiales)*		
Rhizobiaceae		

* Agrobacterium*	11 valid names	2 rhizobia
* Allorhizobium*	8 valid names	2 rhizobia
* Ensifer* (=*Sinorhizobium*)	20 valid names	20 rhizobia
* Neorhizobium*	5 valid names	4 rhizobia
* Pararhizobium*	6 valid names	2 rhizobia
* Rhizobium*	92 valid names	49 rhizobia
* Shinella*	8 valid names	1 rhizobia

Phyllobacteriaceae		

* Aminobacter*	7 valid names	1 rhizobia
* Phyllobacterium*	12 valid names	4 rhizobia
* Mesorhizobium*	59 valid names	48 rhizobia

Nitrobacteraceae (=Bradyrhizobiaceae)		

* Bradyrhizobium*	63 valid names	58 rhizobia

Methylobacteriaceae		

* Microvirga*	18 valid names	4 rhizobia
* Methylobacterium*	47 valid names	1 rhizobia

Brucellaceae		

* Brucella* (=*Ochrobactrum*)	25 valid names	2 rhizobia

Hyphomicrobiaceae		

* Devosia*	26 valid names	1 rhizobia

Xanthobacteraceae		

* Azorhizobium*	3 valid names	2 rhizobia

*β-Proteobacteria*		
Burkholderiales		
Burkholderiaceae		

* Cupriavidus*	18 valid names	2 rhizobia
* Paraburkholderia*	79 valid names	22 rhizobia
* Trinickia*	7 valid names	1 rhizobia

## Abbreviations

ANI: Average nucleotide identity
DDH: DNA-DNA hybridization
dDDH: Digital DNA-DNA hybridization
ERIC: Enterobacterial repetitive intergenic consensus
GDP: Growth domestic product
HGT: Horizontal gene transference
ICNP: International Code of Nomenclature of Prokaryotes
ICSP: International Committee on Systematic of Prokaryotes
IJSEM: International Journal of Systematic and Evolutionary Microbiology
ITS: Internal transcribed spacer
LPSN: List of prokaryotic names with standing in nomenclature
MEGA: Molecular evolutionary genetics analysis
MGE: Mobile genetic elements
MLSA: Multilocus sequence analysis
MLST: Multilocus sequence typing
MYA: Million years ago
NGS: Next-generation sequencing
NI: Nucleotide identity
OGRI: Overall genome-related indices
PCR: Polymerase chain reaction
REP: Repetitive extragenic palindromic.
